# Efficient Classification of Motor Imagery Electroencephalography Signals Using Deep Learning Methods

**DOI:** 10.3390/s19071736

**Published:** 2019-04-11

**Authors:** Ikhtiyor Majidov, Taegkeun Whangbo

**Affiliations:** Department of Computer Science Gachon University, Sujeong-Gu, Seongnam-Si, Gyeonggi-Do 13109, Korea; ixtiyoruz312@gmail.com

**Keywords:** tangent space, Riemannian geometry, particle swarm optimization (PSO), BCI, EEG, electro-oscillography (EOG), CSP, FBCSP (filter bank common spatial pattern), online learning

## Abstract

Single-trial motor imagery classification is a crucial aspect of brain–computer applications. Therefore, it is necessary to extract and discriminate signal features involving motor imagery movements. Riemannian geometry-based feature extraction methods are effective when designing these types of motor-imagery-based brain–computer interface applications. In the field of information theory, Riemannian geometry is mainly used with covariance matrices. Accordingly, investigations showed that if the method is used after the execution of the filterbank approach, the covariance matrix preserves the frequency and spatial information of the signal. Deep-learning methods are superior when the data availability is abundant and while there is a large number of features. The purpose of this study is to a) show how to use a single deep-learning-based classifier in conjunction with BCI (brain–computer interface) applications with the CSP (common spatial features) and the Riemannian geometry feature extraction methods in BCI applications and to b) describe one of the wrapper feature-selection algorithms, referred to as the particle swarm optimization, in combination with a decision tree algorithm. In this work, the CSP method was used for a multiclass case by using only one classifier. Additionally, a combination of power spectrum density features with covariance matrices mapped onto the tangent space of a Riemannian manifold was used. Furthermore, the particle swarm optimization method was implied to ease the training by penalizing bad features, and the moving windows method was used for augmentation. After empirical study, the convolutional neural network was adopted to classify the pre-processed data. Our proposed method improved the classification accuracy for several subjects that comprised the well-known BCI competition IV 2a dataset.

## 1. Introduction

Creating brain–computer interface (BCI) applications based on electroencephalograms (EEG) is a challenging scientific task given that they translate the mental imagery to sets of commands without using any muscles. BCI applications are a valuable part of neuroscience, neural engineering, and medicine, in which robotics or mental issue detectors are used. To date, they have been used extensively in many areas of medicine to help people by connecting their minds to control devices or by detecting brain abnormalities [1,2,3]. This can be recognized by measuring the electric or magnetic fields generated by the central nervous system using electroencephalography (EEG) or magnetoencephalography (MEG) [4]. Electroencephalographic signals are usually recorded with the placement and use of EEG sensors with another kind of electrode placed onto the surface of the scalp using a 10–20 electrode placement system (Jasper 1958).

Frequency-based features have gained significant importance in this area, and various frequency-based feature extraction methods have been proposed [5]. The best-known approach is the power spectrum density approach [5]. It is computed using the classical signal processing algorithm known as the Fourier transform (FT) or the computationally efficient fast Fourier transform (FFT) form. The problem with this approach is that it lacks the capacity to preserve spatial information. 

One of the algorithms used to retrieve spatial information from EEG signals is the common spatial pattern (CSP) algorithm. CSP first appeared in the area of EEG and MEG analyses as used by Koles [6]. It was initially proposed for two different classes, such as for left or right-hand movements [7]. It computes spatial filters which maximize the variance ratio of one of the label conditions with respect to another. The main disadvantage of this algorithm is that it works only with two classes. To solve this problem, several approaches have been proposed, such as the pairwise and the one-versus-rest approaches; however, the main problem of these algorithms is that they use multiple classifiers to obtain the final result. To use only one classifier, we employed the simultaneous diagonalization approach in conjunction with the information-theory-based approach, based on information theoretical feature extraction (ITFE) [4], which uses only one classifier. The detailed explanation of the ITFE method will be discussed in a subsequent subsection.

The presence of noise makes the classification of EEG signals difficult. EEG signals are prone to external and internal noise. Numerous methods have been proposed to reduce noise in EEG signals [8], such as independent component analysis (ICA) [9]. To obtain subject-dependent spatial and frequency-based features, combinations of these features are used. In the filterbank CSP (FBCSP) approach, filterbanks and CSPs were used [10]. 

Recently, Riemannian geometry-based feature extraction and classification methods have gained significant importance in BCI applications. These methods were initially used in BCI applications in [11] and won the first place in the BCI Challenge NER 2015. The same authors used the distance between the covariance matrix and Riemannian mean covariance matrix as a classification feature. Furthermore, Barachant et al. [11] introduced mapping covariance matrices on the tangent space of the Riemannian manifold. Mapped covariance matrices are represented as vectors in the tangent space.

The use of deep-learning-based classification methods in BCI applications is rare, owing to the complexity of the recording and the limited numbers of signals. In [12], Bashivan et al. used power spectrum densities based on three frequency ranges of EEG signals and generated images for each range by interpolating the topological features that preserved the surfaces of the brain. They used the VGG (visual geometry group) model and mixed 1D convolutions and long short-term memory (LSTM) layers. The results showed that the ConvNet and LSTM/1D-Conv yielded the best results compared to other architectures. In [13], the architecture was also based on convolutional neural networks, and the authors used the convolutional layer first and then the encoder part of the AutoEncoder. Additionally, they also used the power spectral densities of the fast Fourier transforms as a feature set. 

In this work, initially, signals were augmented as represented in Section 3.1, filtered with the given filterbanks, and fed to the ITFE-based multiclass CSP algorithm to reject the unimportant parts. Thereafter, the results of the CSP method were concatenated and covariance matrices were computed. Next, the covariance matrices were mapped onto the tangent space of the Riemannian manifold, and the results were concatenated with flattened covariance matrices. Finally, power spectrum density (PSD) features were also concatenated with the last results, and training data was formed. Furthermore, the particle swarm optimization method was used to replace bad features with the linear average values of the neighboring features. Eventually, one layer of the convolutional neural network was implied to classify the pre-processed data.

The rest of this article is constructed as follows: Section 2.1 and Section 2.2 explain the algorithm filterbank CSP (FBCSP), and Section 2.3 and Section 2.4, respectively, explain the power spectrum density and the Riemannian geometry as well as its usage. Section 3 discusses our proposed method where the data augmentation method is presented in Section 3.1, and the filterbank particle swarm optimization (FBPSO) feature-selection algorithm is represented in Section 3.2. Accordingly, datasets and generated results are presented in Section 4, followed by a brief summary of the study. 

## 2. Related Studies

### 2.1. Filterbank Common Spatial Pattern

**Figure 1:**
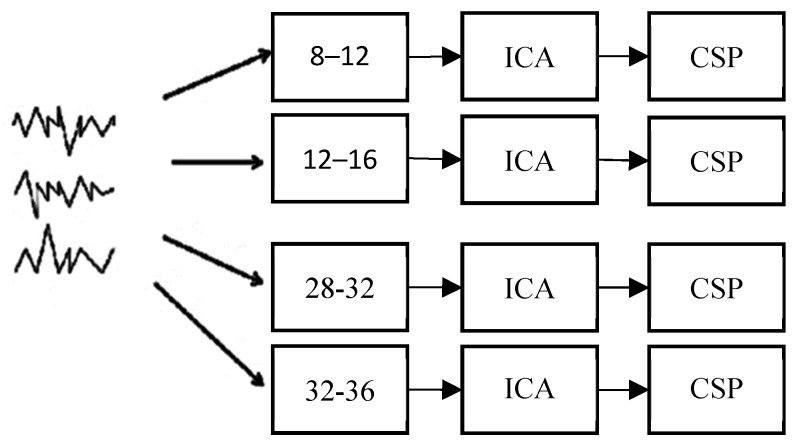
Division of signals into subsignals by filtering followed by the application of spatial filters, as described by [10]. CSP: common spatial pattern.

The FBCSP [10] is shown in Figure 1. The first stage is the bandpass filtering stage that uses ICA decomposition to reduce noise in the second stage. Moreover, the third stage fitted the CSP and formulated the spatial filter, followed by the application of the spatial filters and transformation of the results into CSP spaces.

#### 2.1.1. Filtering at the Base Frequency

Initially, the signal was divided into filterbanks with the help of IIR filters. The cut-off frequencies of the filters were chosen in the range of 8–36 Hz because better results were obtained in this range.

#### 2.1.2. Common Spatial Patterns for Two Classes

The CSP algorithm was then applied after the blind ICA source separation algorithm was deployed because it constitutes one of the best ways to reduce noise in brain signals. The core idea of CSP is the maximization of one class of features and the minimization of another so that the resulting signals encode the most significant information [14]. 

If two conditions, a and b, and the respective matrices X^a^ and X^b^ exist, they define an N × T shape, where N is the number of electrodes, and T is the number of samples per electrode. Firstly, we have to find the normalized covariance of the matrices of the trials:

(1) $$R_{a}^{i}=\frac{X_{i}^{a}X_{i}^{a\;T}}{{\mathrm{trace}(X}_{i}^{a}X_{i}^{a\;T})},$$

where $R_{a}^{i}$ indicates the normalized covariance matrix of the i^th^ trial of a group or a condition “a” (similarly for $R_{b}^{i}$). Subsequently, the normalized covariance matrices are averaged according to

(2) $$R^{a}=\frac{\sum_{i=1}^{n}R_{a}^{i}}{n},{\;R}^{b}=\frac{\sum_{i=1}^{n}R_{b}^{i}}{n},$$

The summation of these allows the formulation of the composed matrix Rc and the estimation of the eigenvalues and vectors, whereby $R^{c}=B_{c}{\lambda B}_{c}^{T}$, and $B_{c}$ is the matrix of eigenvectors. λ is a diagonal matrix of eigenvalues. The whitening transform is computed by $W_{\mathrm{whitening}}=\lambda^{-1/2}B_{c}^{T}$. Let S_a_ and S_b_ be

(3) $$S_{a}={\mathrm{WR}}_{a}W^{T}\;\mathrm{and}\;S_{b}={\mathrm{WR}}_{b}W^{T}$$

In the next step, we identify the aforementioned eigen decomposition of these matrices, which are expressed as

(4) $$S_{a}={U\psi }_{a}U^{T}\;\mathrm{and}\;S_{b}={U\psi }_{b}U^{T}$$

The eigenvalues of the equations in (4) satisfy this equation $\psi_{a}+\psi_{b}=I$. Eventually, our spatial filter will be computed according to

(5) $$P^{T}=U^{T}\;W,$$

Thus, this filter can be applied as

(6) $$X_{f}^{i}=X^{i}{\;P}^{T},$$

Our P matrix then satisfies these equations according to the representation listed in [15],

(7) $$P^{T}R_{a}P=D_{1},$$

(8) $$P^{T}R_{b}P=D_{2},$$

which means that the spatial filter P diagonalizes both covariance matrices.

After filtering the data, we can save only the important features and discard the less informative ones. To do this, m/2 rows above and m/2 rows below the matrix are selected so that information that represents both conditions is maintained. In the end, an m × T matrix is constructed, where m is the number of selected rows.

**Figure 2:**
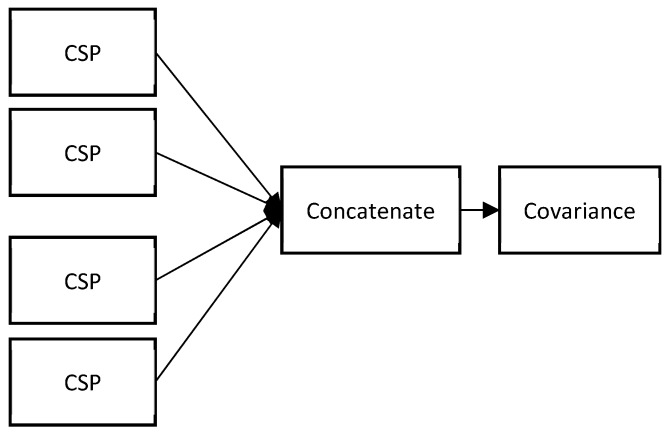
Graphical representation of the process of concatenation.

In this study, after the completion of the filtering process, we had seven spatially filtered filterbanks with sizes of m × T, where m = 4 and T = 500 in this case. These filterbanks are concatenated, as represented in Figure 2. The axis of zero is used for concatenation so that the resulting matrix has the form of (m × 7) × T; that is, 42 × 500. Accordingly, the computation of the covariance matrix based on this formulation yields a 36 × 36 matrix.

### 2.2. Multiclass Filterbank Common Spatial Pattern

The main objective associated with the use of spatial filters is to identify a signal’s inner space with given conditions (classes). In this way, the CSP algorithm identifies one inner space whereby conditions are maximally represented. When multiple conditions exist (more than two), the problem is complex and difficult to handle. In [4], the information theoretic feature selection (ITFE) was proposed based on the joint approximate diagonalization (JAD) algorithm that solves minimal achievable classification and multiclass problems. It is based on the maximization of mutual information between data X and gives the labels $P^{*}=\mathrm{argmax}\left\{{I(c,\;P}^{T}R)\right\}$ so that L rows from the data matrix X can be selected as a signal’s inner space, which preserves most of the information. The implementation sequence of the algorithm is shown below.

First, the covariance matrices need to be computed as $R_{{x|c}_{i}},i=1,\;\ldots ,\;M$, where M is the number of classes. 

Subsequently, the JAD algorithm has to be deployed. It is based on Equations (7) and (8), and as stated in [16], for every covariance matrix belonging to M classes, a W matrix has to be identified which diagonalizes all the covariance matrices as $W^{T}R_{{x|c}_{i}}W=D_{c_{i}},\;i=1,\;\ldots ,\;M$. 

Next, every column of $w_{j},\;j=1,\;\ldots ,\;N,\;\mathrm{of}\;W$ where $w_{j}$ is the $j_{\mathrm{th}}$ column of matrix W is taken and changed so that it satisfies $w_{j}^{T}R_{{x|c}_{i}}w_{j}=1$; then, the mutual information is computed according to the equation below (9) [4]:

(9) $${I(c,\;w}_{j}^{T}x)\approx -\sum_{i=1}^{M}P\left(c_{i}\right)\log\sqrt{w_{j}^{T}R_{{x|c}_{i}}w_{j}}-\frac{3}{16}{\left(\sum_{i=1}^{M}P\left(c_{i}\right)\left({\left(w_{j}^{T}R_{{x|c}_{i}}w_{j}\right)}^{2}-1\right)\right)}^{2},$$

Eventually, L columns of W have to be selected and applied to the data X using the dot product.

### 2.3. Power Spectrum Density (PSD)

Simultaneously, the PSDs of all frequency bands were computed, including the mu (8–12 Hz), beta (13–25 Hz), and gamma (30–45 Hz) bands, with the measurement of the FBCSPs of signals and their concatenations, as for CSPs above. Some EEG headsets provide the PSDs automatically, and for convenience it is thus helpful to have a dataset that has this property. 

### 2.4. Riemannian Geometry

The fundamental idea of this geometry is based on the mapping of the covariance matrix on the space which conveniently represents the data. The method learns the curve-shaped spaces that comprise Euclidean spaces. Therefore, as with the surface of the earth, the covariance matrix is located on a curve-based-space, and one should approach this accordingly. In the BCI field, it is assumed that the existence and use of the EEG signal are located in the specific curve-shaped space. Accordingly, if the signal is mapped to the defined space, it can be used more effectively. In this study, the concepts of Riemannian geometry were described, and the reader’s attention is redirected to [11] and to the references listed therein for further instructions.

#### 2.4.1. Spatial Covariance Matrices

Covariance matrices are computed using the equation given in (10). Suppose the original recording of the EEG signal is stored in matrix X, which has a shape of N × T, where N is the number of channels and T is the number of samples. In the calibration mode, each EEG signal is divided into supervised segments called trials with sizes of N × $T_{s}$, where $T_{s}$ denotes the number of samples per trial. To apply it into Riemannian geometry-based algorithms, it should satisfy the equation $T_{s}\gg N$, and it should be symmetric positive–definite (SPD), which means it must be diagonalized with real positive eigenvalues [11].

(10) $$C_{i}=\frac{X_{i}X_{i}^{\;T}}{N},$$

#### 2.4.2. Spatial Covariance Matrices

Let us consider that one has a set of matrices P which have m × m dimensions. As represented in [17], this set can be defined as $P(m)=\{P\in S\left(m\right){|u}^{T}\mathrm{Pu}>0,\;\forall u\in {\;R}^{m},\;u\neq 0\}$, where S(m) indicates the space of all symmetric matrices. Moreover, this creates a manifold ℳ with the dimension m(m+1)/2. 

The geodesic distance denotes the minimum length of the path between two points on the manifold ℳ. The geodesic distance between two SPD covariance matrices can be estimated using the equation below:

(11) $$\delta_{R}\left(P_{1}{,P}_{2}\right)={\|\log(P}_{1}^{-1}P_{2}){\|}_{F}={\left[\sum_{i=1}^{n}\log^{2}\lambda_{i}\right]}^{1/2},$$

where $\lambda_{i},\;i=1,\;\ldots ,\;n$ indicates the real eigenvalues of $P_{1}^{-1}P_{2}$. 

#### 2.4.3. Approximation of SPD Matrices

In Riemannian geometry, the mean of n ≥ 1 SPD matrices, which is also referred to as the geometry mean, can be formulated based on the geodesic distance:

(12) $$Q_{\mathrm{mean}}\left(P_{1}{,\;P}_{2}{,\;\ldots ,\;P}_{n}\right)=\underset{P\in P(m)}{\arg\;\min}\sum_{i}^{N}{\delta_{R}}^{2}\left({P,P}_{i}\right),$$

#### 2.4.4. Tangent Space Mapping

Barachant et al. [11] also proposed a method which maps the covariance matrices in a tangent space of the Riemannian manifold. Each SPD matrix P∈P(m), which is a point in Riemannian geometry, has a mapped version in the tangent space with the same dimension as that of the manifold m(m+1)/2. The tangent space mapping is computed using the following equation:

(13) $$S_{i}=\mathrm{upper}(\log({Q_{\mathrm{mean}}}^{-\frac{1}{2}}P_{i}{Q_{\mathrm{mean}}}^{-\frac{1}{2}})),$$

**Figure 3:**
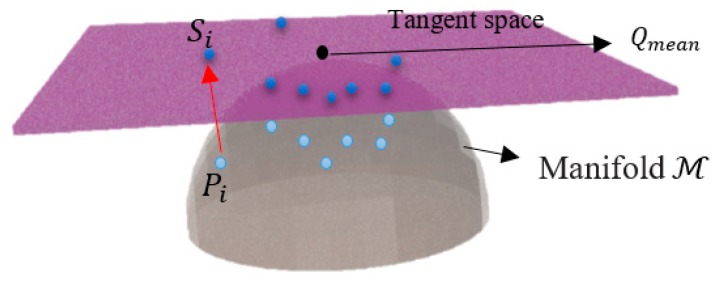
Graphic example of tangent space mapping, whereby the red arrow represents the exponential mapping of S_i_.

As shown in Figure 3, each of the SPD matrices map onto the tangent space at point $Q_{\mathrm{mean}}$, which is a point calculated with the use of Equation (12) and vectorized by obtaining only the triangular part of the matrix that corresponds to the dimensions of the tangent space.

### 2.5. Particle Swarm Optimization

Particle swarm optimization (PSO) is an optimization method that works like a genetic algorithm (GA) [18]. However, it is easier and can be implemented with a few lines of code. PSO originated from the traversing of flocks of birds and advances a problem by trying to improve the candidate solution in a continuous manner, based on a given quality ratio [19]. 

**Algorithm 1.** Simple particle swarm optimization (PSO) pseudocode.1. **begin** 2. initialize 3. **for** i **in** n_iterations = k 4.   **for each** position p **of** particle compute fitness 5.     **if** fitness (p) > fitness (pbest) 6.       pbest = p 7.   set best of pbest as gbest 8.   update particles velocity and position 9. gbest is our result

The uniqueness of the algorithm compared to GA is based on the fact that it stores the variables for each particle’s personal best position (pbest), global best position (gbest), velocity v, and current position x. As shown in the pseudocode of Algorithm 1, the individual best positions can be identified for each particle, and the best one is selected among all the pbest values of all the particles. In the long run, the velocity and the position of each particle can be updated using equation [19]:

(14) $${v=\mathrm{wv}+c}_{1}\mathrm{rand}()\left(\mathrm{pbest}-x\right)+c_{2}\mathrm{rand}()(\mathrm{gbest}-x),$$

(15) x=x + v,

where w indicates the inertia value that represents the percentage of the old velocity value which will be maintained, and $c_{1}\in \left[0,\;1\right]$ and $c_{2}\in \left[0,\;1\right]$ are known as the “acceleration coefficients” used for the selection of the pbest and gbest values, respectively. The function rand() generates random (i.e., different) values between zero and one.

## 3. Proposed Method

**Figure 4:**
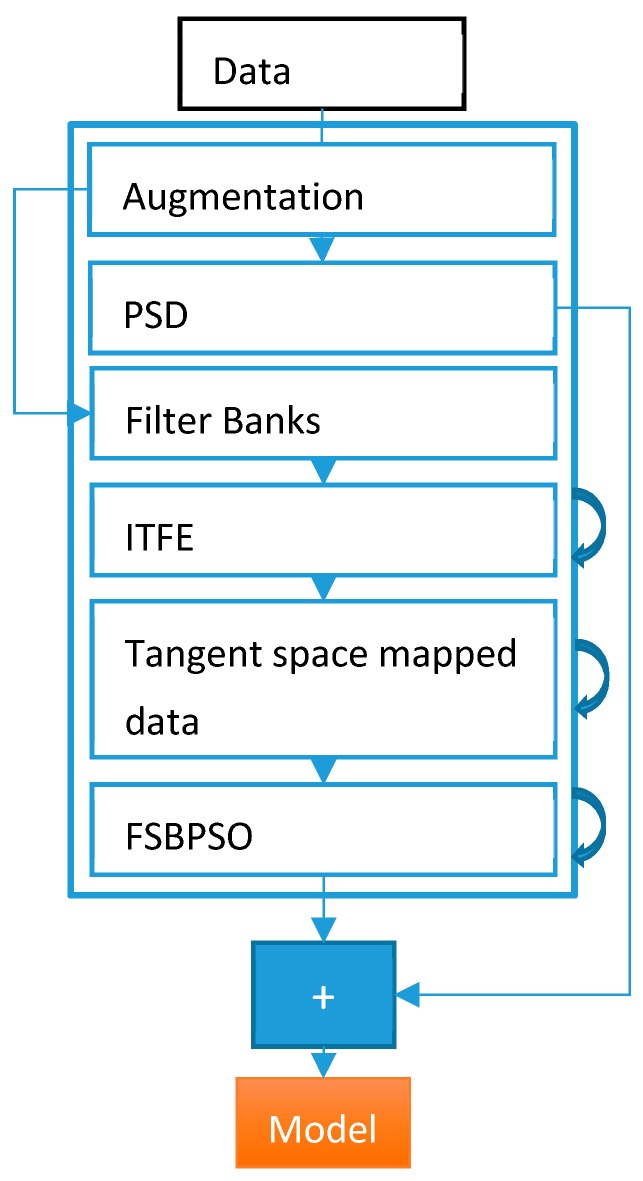
Overall architecture of the proposed method, where the curved arrows represent the algorithms which have two modes; that is, for training and testing.

In this study, a number of remarkable algorithms were used. After the data was read, data augmentation was applied. The augmentation process was completed by moving the sliced windows described in Section 3.1. The general architecture of the proposed method is depicted in Figure 4. 

### 3.1. Data Augmentation

**Figure 5:**
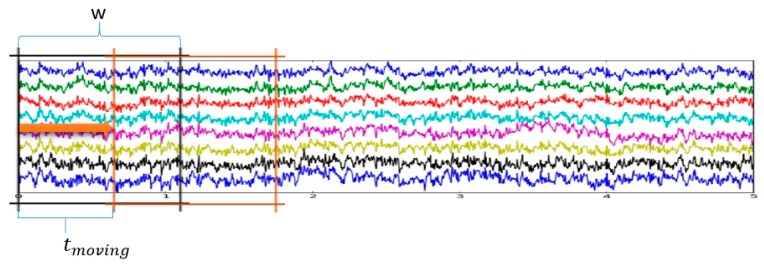
Graphical illustration of the augmentation process.

For data augmentation, the sliced windows method with a window size of w and a moving time of $t_{\mathrm{moving}}$ was used. At each time instant, the window w was used such that there was a specific moving time $t_{\mathrm{moving}}$, as represented in Figure 5.

### 3.2. FBCSP Algorithm

In this study, first, the filtering operation was used, as shown in Section 2.1, after the independent component analysis was adopted to reduce the noise of the data.

After the noise reduction, the CSP algorithm was deployed, as shown in Section 2.1 and Section 2.2, and m rows of the spatially filtered data were selected. As mentioned earlier, they were concatenated. Suppose $n_{f}$ frequency bands and $n_{t}$ samples were selected in each time trial. Overall, $n\times \left({m\times n}_{f}\right){\times \;n}_{t}$ shaped data were taken, where n is the number of trials. The next step was the computation of the covariance matrices of the trials using the equation given in (10). Eventually, the data size was be $n\times \left({m\times n}_{f}\right)\times \left({m\times n}_{f}\right)$, whereby the data comprises n square matrices.

### 3.3. PSD Algorithm

At the same time, the PSDs of the data were computed for the $n_{f}^{p}$ frequency bands using three frequency bands; i.e., alpha, beta, and gamma. The dataset $D_{\mathrm{psd}}$ obtained from the use of this algorithm had a size of ${n\times (N}_{\mathrm{ch}}n_{f}^{p})$.

### 3.4. Tangent Space Mapping

After the completion of all the calculations indicated above, two types of data composed of covariance matrices and power spectral densities were retrieved.

The next step in this process was the deployment of the tangent space mapping explained in Section 2.4. The result was the vector D_ts_^i^, i ∈ 1, …, n, where n is the number of data samples (trials) given in our dataset with a dimension of $\left({(m\;n}_{f}\right)\left({m\;n}_{f}+1)\right)/2$. Subsequently, for each trial, the tangent space ${D_{\mathrm{ts}}}^{i}$ and the flattened covariance matrix ${D_{c}}^{i}$ were concatenated. Consequently, the $D_{\mathrm{temp}}$ matrix with the shape of $n\times \left({\left({m\;n}_{f}\right)}^{2}+\left({m\;n}_{f}\right)\left({m\;n}_{f}+1\right)/2\right)$ was obtained. Correspondingly, when the subsequent part was replaced with $n_{\mathrm{new}}$, the shape of $D_{\mathrm{temp}}$ was ${n\times n}_{\mathrm{new}}$. 

### 3.5. FSBPSO Algorithm

**Figure 6:**
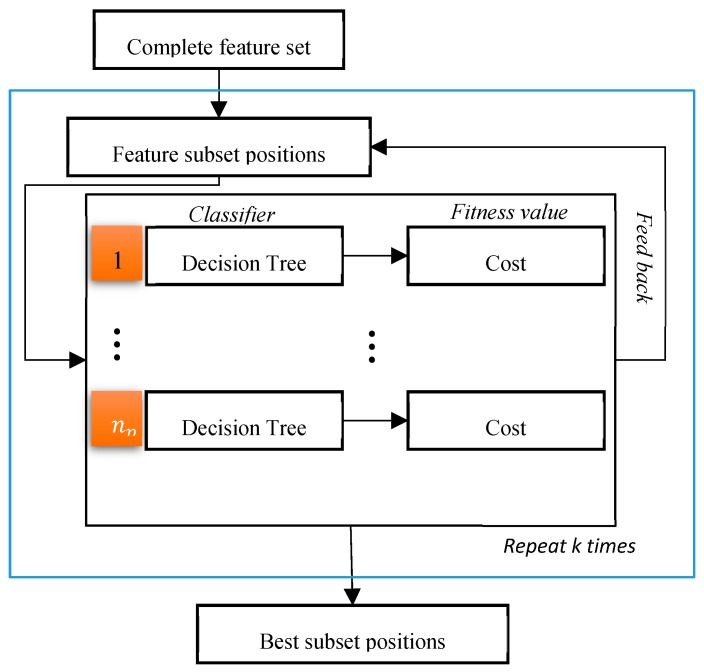
Graphical illustration of the filterbank particle swarm optimization (FBPSO) algorithm, where $n_{p}$ is a number of particles for PSO.

In our study, the wrapper-based feature-selection algorithm called feature selection with the binary PSO algorithm FSBPSO was deployed. A similar approach was adopted in [20]. It is obvious from its name that FSBPSO is a feature-selection algorithm based on PSO optimization. As represented in Figure 6, the entire dataset was input at the beginning, and the FSBPSO initialized positions randomly by

(16) $$X_{i,j}=\{\begin{array}{l} 1,\;\mathrm{if}\;\mathrm{the}\;\mathrm{feature}\;\mathrm{selected}\\ 0,\;\mathrm{otherwise} \end{array},$$

where j = $j\in {1,\;\ldots ,\;N}_{\mathrm{new}}$, represents the number of features. Subsequently, based on the quality value, which determines the cost accuracy taken from the KNN algorithm, the pbest and gbest values were found, and the position and velocity values were updated based on Equations (14) and (15). At the end, a vector with a shape of $N_{\mathrm{new}}$ that will consist of ones and zeros according to Equation (16) was achieved. 

At this stage, the positions of the features which best defined the data were retrieved. Herein, this feature selection was applied on the data $D_{\mathrm{temp}}$, and a better accuracy than the concatenated case with $D_{\mathrm{psd}}$ was obtained. During the feature selection, the data with a linear interpolation scheme was interpolated to fill the missing gaps. This means that the non-selected features were effectively replaced with average values. Subsequently, to obtain the final data D, the feature selections of $D_{\mathrm{temp}}$ and $D_{\mathrm{psd}}$ were concatenated. Therefore, the final data D had shape dimensions equal to $n\times \left(n_{\mathrm{new}}+N_{\mathrm{ch}}n_{f}^{p}\right)$. The last part was set to $n_{\mathrm{final}}=n_{\mathrm{new}}+N_{\mathrm{ch}}n_{f}^{p}$ so that in the end, D had shape dimensions equal to ${n\times n}_{\mathrm{final}}$.

### 3.6. Architecture

The use of deep networks is very rare in the BCI field owing to the increased noise ratio and the lack of adequate data. In this study, the number of training samples or signal trials was improved using data augmentation, as mentioned earlier. The application of a number of large and deep networks has also been attempted. However, as the networks became deeper, overfitting increased. Thus, using very deep networks was stopped. Instead, to classify the data D, a 1D convolutional neural network (CNN) was used. It is not uncommon for CNNs to be used in the classification of EEG signals. In [21] and [12], 1D convolutional networks were used in combination with long short-term memory (LSTM). In the first case [21], the evoked results revealed that the combined architecture just outperformed the CNN. However, when they used only LSTM, the result was poor in comparison to the CNN. In [13], the authors used stacked CNNs as the encoder part of the AutoEncoder with the FFT features on two frequency bands: i.e., the mu (8–13Hz) and beta (13–30 Hz) bands. 

In this study, two types of architectures were used. The first one was composed of CNN and output softmax layers only. In the second one, the CNN layer was followed by fully connected layers with 100 output units. 

For training, the filter size was set to seven and the activation functions to rectified linear unit. To optimize our model, the Adam optimizer was used, and the learning rate applied varied based on the dataset and subjects. The number of epochs for training was set to 20. Correspondingly, increasing this number led to overfitting.

## 4. Experiments and Results

### 4.1. Datasets

To show how well our algorithm works, two publicly available datasets, 2a and 2b from the BCI Competition IV, were selected. Both of them were mental imagery datasets, and both included left- and right-hand movements. These datasets were recorded during the imaginary movement of hands or feet and were sampled at a 250 Hz frequency rate. For detailed information, please refer to [22]. 

The dataset 2a consists of 22-channel data from nine subjects and was presplit into training and testing sets. However, dataset 2b comprised nine subjects with three channels of EEG signals.

### 4.2. Results

In this study, experiments have been conducted with the use of the Python 3.6 environment with the MNE–Python EEG signal processing tool on an Intel (maximum 4.7 GHz) core i7 PC with 16 GB of RAM. The training and testing of each dataset was conducted separately for each subject. In the analysis process for dataset 2a, for example, the first subject’s training and testing process was completed on the A1T and A1E files, respectively. Herein, accuracies are listed only for the test set. 

The results of dataset 2a are listed in Table 1. These values have been obtained using semi-supervised online learning methods, based on which our model was trained using predicted test data labels.

In the data augmentation process represented in Section 3.2, the number of iterations was four, the moving time was 0.3 s, and the window size w was 3 s. In the spatial filtering process, the number of selected rows m was six. The parameters of our FSBPSO algorithm during the stage of feature selection are listed in Table 2. 

For comparison, the results from several prior publications were distinguished based on state-of-the-art approaches. It is clear from the table that our method has comparable results with other methods (sample size = four subjects). If the average values of the results were taken into consideration, one can see that our proposed method surpassed the majority of the other methods by reaching a classification accuracy of 80.44%, as shown in Table 1. The authors of SR-MDRM used a very similar approach to our method; however, in their work, a spatial filter regularized by coordinates of electrodes to ensure prior information from EEG channels and a minimum classification distance to the Riemannian mean was used. Furthermore, In WOLA-CSP [26], the WOLA algorithm, which consists of applying FFT to compute power spectrum density, shifting the spectrum of given frequencies measured by ERDSA, which is a specific-subject frequency selection algorithm, to the origin of the axis, and subsequently using IFFT to transform the spectrum back to the time domain, was used. Additionally, CSP to extract features and latent Dirichlet allocation (LDA) to classify the retrieved features were used; however, the data were still sparse.

In addition to this, the results obtained from the next dataset 2b are listed in Table 3. The dataset contained highly corrupted EEG signals, but the ICA algorithm managed to filter the signal. Data augmentation was conducted based on the same procedure as that used for the dataset 2a.

The features of the dataset have been obtained by the proposed algorithm, and the parameters of the FSBPSO algorithm are also the same as those for dataset 2a, as listed in Table 2.

To compare the results of dataset 2b, the feature set was evaluated with other well-known machine learning techniques, including the latent Dirichlet allocation (LDA) and support vector machines, and constructed similar encoder parts of the AutoEncoder with CNNs based on the formulation of the CNN–SAE model of Yousef and Ugur [13]. As shown, our method yields better results for all the tested subjects except for Subject 4, and the average value of the classification accuracy (82.39 %) also outperforms the corresponding accuracies of the other studies, as indicated in Table 3.

The comparisons listed above were for two-class cases only; that is, for the right and left hands. For multiclass cases, 10-fold cross-validation classification results for the 2a dataset from the BCI competition IV [27] are represented for all the classes, including the left hand, right hand, tongue, and both feet, as shown in Table 4.

For comparison, three related results for the dataset are represented, including the TSLDA [11], which represents the tangent space mapping feature extraction method alongside the LDA classification method, and the CSP* and the LDA method, in which the best features were selected for each subject according to the FDR criterion [11] in association with the LDA classification method. The results for the MDRM minimum distance to Riemannian manifold method [11] were also presented. Our method outperformed all the referenced examples, including the latter one.

## 5. Discussion and Conclusions

In this study, a number of well-known feature extraction methods were combined for EEG signal processing, and one of the deep-learning-based approaches was described. In addition to this, several deep learning techniques were also evaluated, such as online learning (unsupervised learning) and transfer learning. It was found that by using online learning, one could increase the classification accuracies of the EEG signals by almost 2%, while transfer learning led to bad results. It was observed that very deep networks can cause overfitting. However, if any one layer in the CNN network does not perform well, the parameters of the model are not strong enough to allow the learning of the problem. Therefore, in instances where this occurred, another fully connected layer was added immediately after the layer with poor performance. Deep-learning-based techniques often require an extensive number of resources. However, in this study, this problem was solved with the use of data augmentation.

## Figures and Tables

**Table 1:** Comparison of classification accuracy (in %) for the completion of the LvR task of the proposed method with published results of the dataset 2a obtained from the BCI competition IV that included left- and right-hand movement data.

Subject	Proposed Method	Raza et al. [23]	Gramound Gaur. [24]	SR-MDRM [25]	WOLA-CSP [26]
A1	90.14%	90.28%	**91.49%**	90.21%	86.81%
A2	62.35%	57.64%	61.27%	63.28%	**63.89%**
A3	89.17%	95.14%	94.89%	**96.55%**	94.44%
A4	**79.23%**	65.97%	76.72%	76.38%	68.75%
A5	**76.45%**	61.11%	58.52%	65.49%	56.25%
A6	**69.35%**	65.28%	68.52%	69.01%	69.44%
A7	**84.08%**	61.11%	78.57%	81.94%	78.47%
A8	86.10%	91.67%	**97.01%**	95.14%	97.91%
A9	87.06%	86.11%	**93.85%**	93.01%	93.75%
Average	80.44%	74.92%	79.93%	**81.22%**	78.86%

**Table 2:** Parameters for the FSBPSO algorithm.

$c_{1}$	$c_{2}$	w	$n_{p}$	K
0.9	0.8	0.7	8	200

**Table 3:** Comparison of the classification accuracy (in %) with other machine learning techniques based on the dataset 2b obtained from the BCI competition IV.

Subject	Proposed Method	LDA	SVM	Yousef and Ugur [13]
1	**78.81%**	64.58%	74.65%	76.00%
2	**80.14%**	58.08%	75.36%	65.80%
3	**77.08%**	61.11%	70.13%	75.30%
4	91.37%	67.34%	81.98%	95.30%
5	**86.15%**	66.55%	79.10%	83.00%
6	**80.73%**	59.72%	72.91%	79.50%
7	**81.59%**	69.79%	80.55%	74.50%
8	**81.25%**	67.10%	76.64%	75.30%
9	**84.37%**	60.06%	77.08%	73.30%
Average	**82.39%**	63.81%	76.49%	77.56%

**Table 4:** Comparison of cross-validation classification results (in %) with other related approaches for the 2a multiclass dataset obtained from the BCI competition IV.

Subject	Proposed Method	TSLDA	CSP*+LDA	MDRM
1	**93.30%**	80.50%	81.80%	77.80%
2	**84.59%**	51.30%	45.10%	44.10%
3	**91.68%**	87.59%	83.50%	76.80%
4	**84.55%**	59.30%	59.00%	54.90%
5	**86.54%**	45.00%	42.20%	43.80%
6	**76.92%**	55.30%	43.30%	47.10%
7	**94.03%**	82.10%	81.50%	72.00%
8	**93.20%**	84.80%	69.60%	75.20%
9	**92.24%**	86.10%	80.00%	76.60%
Average	**87.94%**	70.20%	65.11%	63.20%
